# Filling Teeth

**Published:** 1866-06

**Authors:** J. Taft


DENTAL REGISTER.

VOL. XX.] JUNE 1866. [NO. 6.

### Original Communications.

### FILLING TEETH.

BY J. TAFT.

It might seem, at first thought, that the subject of filling teeth had been completely exhausted—the whole ground thoroughly canvassed, considered, described and illustrated.

There has not, however, been much written on this subject for two or three years; this, perhaps, occurs from the supposition that there is little or no progress being made in this operation, such a supposition, however, is far from being correct; there *is* great progress being made, it is shown in two aspects. And first, there is in the profession a general and rapid diffusion of the most approved methods of filling. Many in the profession have, within a short time, made such advances as compel them to admit that the former methods and knowledge were comparatively of little worth. There is a continual progress being made in almost every particular in this operation.

Again, with those who have been regarded as occupying the front rank, there is a continual advance; perfection is not yet attained by any. Which of these two is the more important we will not now attempt to determine, but shall rest satisfied when both go on unobstructed. Progress in this operation, is usually made by an almost imperceptible attainment of things apparently small; these however, in time, being aggregated, revolutionize old methods. In the ordinary plain cases of filling, even those at which the great majority suppose themselves adapts, there is by the present modes almost an entire revolution. A few years ago, the property of cohesion in gold foil, rendered it worthless as a material for filling teeth; now, in the hands of many, it is regarded as indispensible, and to secure it to the greatest degree is a much desired desideratum.

Filling instruments, too, have passed through successive changes, so that now, those of ten or twelve years ago would be regarded as wholly wanting in adaptation. The change in these has been very gradual, and so slow as to be almost imperceptible; yet, let observation extend through ten years, and how marked the change. The great majority of such changes and improvements have been made without any very minute description of them having been made public, either by the pen or tongue, Such things are usually brought into general notice incidentally.

We propose, here, only to notice two or three things in regard to filling teeth, having more particular reference to those cases usually regarded difficult. There are cavaties, large and superficial it may be, and so situated that some operators, and even good ones too, find it difficult to introduce fillings that will be retained. In all such cases there is, on the part of the operator, either carelessness or a want of knowledge and skill, in reference to forming cavities. There is no cavity liable to occur in the teeth, that is not susceptible of being so formed as to retain, firmly, a filling properly introduced. In any cavity, the grooves and anchorages necessary to secure this end, may be made by the diamond point excavators, and the square edged drills. In speaking of fillings that are not retained, we have reference to those in which fillings are disdisplaced because of insufficient anchorage, and not of those fillings which have been lost by additional decay: an example will illustrate.

Recently, a girl twelve years of age, had the left superior lateral incisor filled, on its anterior proximal surface, medium sized cavity, easy of access, with material enough for perfect anchorages, with gold, five times in four months, by an operator who has had a large reputation; and after the filling had fallen out the fifth time, every side and wall of the cavity was converging and smooth, not a vestage of undercut or anchorage any where. In the intense perseverance manifested to succeed, we were reminded of the man who engaged to throw his companion across the river.

For the secure attachment of very large fillings, where a portion of the tooth is broken away, and the part to be built up, or where a large filling is exposed to great pressure in mastication, to insure permanency, in addition to the pits and grooves already mentioned, slits or dove-tails, one or more may be made in the standing portion of the tooth, at such points and in such directions as will not weaken it, these are perfectly filled by anchorages from the main filling, and if properly employed, are of incalculable value in many cases; their greatest advantage, perhaps, is where the entire crown surface is to be added.

And just here another point is presented. It sometimes occurs in large crown cavities of molars and bicuspids, that the edge of the wall is thin and liable to be broken, if not in the operation, in the act of mastication; such edges are often left, the operator filling up to them as best he can, hoping they may stand and do good service; but in the great majority of cases, the tooth will either break away in fragments, leaving the surface rough and unpleasant to the patient, and serves as a lodging point for the foreign substances. In such cases the thin edges should be filed down, from one-sixteenth to one-eighth of an inch, the filed surface having an outward bevel, so that the gold, when built over it, may have a firm embrace upon it, and protect it most completely. The cavity is filled as usual, the gold built up, restoring the original form, and articulating surface of the tooth; by such method there is no comminuting or fretting away of a friable or weak wall, under ordinary pressure upon the filling, but the tooth is held the more firmly together.

(To be Continued.)

